# The poly(A) polymerase PAPS1 mediates pollen maturation by regulating sperm cell differentiation in plants

**DOI:** 10.1002/pld3.397

**Published:** 2022-05-12

**Authors:** Iftikhar Ali, Hassan Sher, Zahid Ullah, Ahmad Ali, Javed Iqbal, Wei‐Cai Yang

**Affiliations:** ^1^ State Key Laboratory of Molecular Developmental Biology, Institute of Genetics and Developmental Biology Chinese Academy of Sciences Beijing China; ^2^ Centre for Plant Science and Biodiversity University of Swat Charbagh Pakistan; ^3^ Department of Botany Bacha Khan University Charsadda Pakistan; ^4^ The College of Advanced Agricultural Science The University of Chinese Academy of Sciences Beijing China

**Keywords:** *Arabidopsis*, cell fate, PAPS1, pollen maturation, sperm cell differentiation

## Abstract

In flowering plants, a haploid microspore undergoes an asymmetric division to produce the male germline that encounters a mitotic division to produce two germ cells. The resulting germ cells undergo a series of specialization events to produce the two sperm cells required for double fertilization. These events include to upregulate male germline‐specific while downregulating male germline‐nonspecific regulon, but how these specializations events are regulated, are still unresolved. To know how plant sperm cell is specialized, we mutagenized *Arabidopsis* double homozygous transgenic line (*MGH3p‐MGH3::eGFP* and *ACTIN11p‐H2B::mRFP*) by an ethyl methane sulfonate (EMS) treatment and isolated a mutant with sperms identity loss, resulting in a completely male defective plant. Second‐generation sequencing identified a point mutation G/A causing premature stop codon TGG/TGA in the poly(A) polymerase PAPS1 that is linked with phenotype. Further, we found that *paps1* mutant fails to upregulate male germline‐specific regulon and to downregulate male germline‐nonspecific factors required for sperm cell differentiation and attaining pollen maturation. Previously, polyadenylation of pre‐mRNAs by PAPS1 has been found crucial for both RNA‐based silencing processes and the processing of pre‐mRNAs into mature mRNAs ready for translation. This study concludes that PAPS1 mediates sperm cell differentiation through upregulating specific while silencing the nonspecific factors of male germlines.

## 1 INTRODUCTION

In flowering plants, the male gametophyte or pollen grain generates and delivers the male gametes to the embryo sac for double fertilization and thus plays a vital role in plant fertility and crop production. Haploid unicellular microspores are produced during male gametogenesis through meiosis within specialized male reproductive organs, the stamens. Each microspore undergoes a highly asymmetric division to produce a small germ or generative cell encapsulated by large vegetative cell. The generative cell then undergoes a second round of mitotic division to create two sperm cells. Sperm cells are most divergent of all cell types as they accomplish their task outside of the male producing body in completely new environment of female body. One sperm cell fertilizes the central cell to produce the endosperm, and the other fertilizes the egg cell to form the embryo. Thus, the making of fully specialized sperm cells is critical for double fertilization and has significant consequences for seed production and crop fertility (Borg et al., 2009; Borg & Twell, 2010).

However, the successful fertilization needs the germ cells to undergo a series of differentiation events to attain fully mature pollen (Borg et al., 2011), but how these events are controlled remain unknown. In previous studies, five pollen‐specific MIKC MADS box proteins expressed in the vegetative cell were found important in pollen maturation (Verelst et al., 2007a, 2007b). Armadillo BTB *Arabidopsis* protein 1 (ABAP1) regulates pollen differentiation by making a potential interaction with a transcription factor TCP16 (Cabral et al., 2021). Further, the distinct and diverse transcriptome of the plant male germ cells enlightening that germline development is transcriptionally tightly regulated (Borges et al., 2008; Engel et al., 2003). However, in terms of male gamete functional specification, only the male germline‐specific R2R3 MYB transcription factor DUO1 pollen1 (DUO1) has been found functioning in the regulation of sperm cell differentiation in *Arabidopsis* (Borg et al., 2011). Here, we found the new and significant role of poly(A) polymerase PAPS1 in mediating pollen maturation by activating a germline‐specific differentiation program through downregulating male gamete nonspecific regulon and upregulating male gamete specific factors.

The poly(A) tail at the 3′ end (polyadenylation) of pre‐mRNAs by PAPS is a critical process in eukaryotic gene expression for both the RNA‐based silencing processes, such as RNA‐directed DNA methylation and the processing of pre‐mRNAs into mature mRNAs ready for translation (Hunt, 2008; Millevoi & Vagner, 2010; Zhang et al., 2019). The *Arabidopsis thaliana* genome encodes four PAPS proteins, termed PAPS1 to PAPS4, and all of them are essential for plant growth and development because homozygous transfer DNA (T‐DNA) insertion mutants could not be obtained for any of the *PAPS* genes (Meeks et al., 2009). The *paps1* mutant produces a male gametophytic defect phenotype and exhibits opposite effects on flower and leaf growth with increased flower growth and reduced leaf growth because of reduced activity of the small auxin‐up RNA (SAUR) (Vi et al., 2013; Zhang et al., 2019). It was demonstrated that the opposite effects of PAPS1 on flower and leaf growth indicate the different identities of these organs and recognize a role for PAPS1 in the vital connection between growth pattern and organ identity. Furthermore, *paps1–3* null‐mutant results in depletion of 21‐ and particularly 24‐nucleotide‐long short interfering RNAs (siRNAs) and microRNAs (miRNAs), a strong overaccumulation of transposable element (TE) transcripts, and defects in the expression of pollen‐differentiation genes. Moreover, PAPS1 was found to regulate pollen development by making a functional interaction with components of mRNA processing and RNA‐directed DNA methylation (RdDM) pathways (Zhang et al., 2019). However, the potential role of PAPS1 in the regulation of sperm cell differentiation and subsequent attaining of pollen maturation remained undetermined. Here, we found that PAPS1 mediates pollen maturation by regulating sperm cell differentiation in *Arabidopsis*.

**FIGURE 1:**
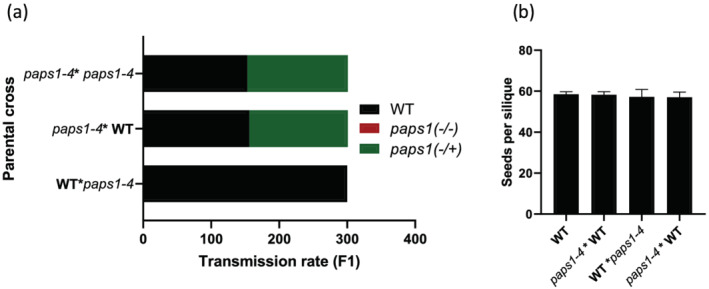
*paps1–4* is male gametophyte defective. Genetic crosses show that *paps1–4* is completely male defective mutant (a). The comparison of numbers of seeds per silique produced in WT and *paps1–4* shows that *paps1–4* does not affect female gametophyte (b). WT = Col‐0

To understand sperm differentiation mechanism in plants, we mutagenized by an ethyl methane sulfonate (EMS) treatment an *Arabidopsis* double homozygous transgenic line *MGH3p‐MGH3::eGFP* and *ACTIN11p‐H2B::mRFP* which is strongly expressed in sperm cells (SC) and vegetative nucleus (VN), respectively. We isolated a mutant (*paps1–4*) with unspecialized sperm cells, resulting in a completely male defective plant (Figure 1). A link between pollen sterility and fluorescence fluctuation in the morphological normal tricellular pollen shows a failure in cell identity in this mutant. The possible reason might be the disruption in the regulation of some of the pollen‐specific genes. Second‐generation sequencing identified a single point mutation G/A linked with this phenotype, causing premature stop codon TGG/TGA in the poly(A) polymerase PAPS1 (AT1G17980), which was confirmed through complementation analysis. Sequencing mutation regions of one thousand autocross plants show that it is completely incredible to get mutated peak in homozygous state presenting complete sterility which is consisted with phenotype.

## 2 MATERIALS AND METHODS

### 2.1 Plant materials and growth conditions

For all experiments, *A. thaliana* ecotype Columbia‐0 (Col‐0) obtained from ABRC stock center (http://www.arabidopsis.org) was used as the wild type. The double transgenic line *MGH3p‐MGH3::eGFP* and *ACTIN11p‐H2B::mRFP* described previously (Borges et al., 2012) was used as control. In mature pollen, *MGH3p‐MGH3::eGFP* is expressed specifically in sperm cells, whereas *ACTIN11p‐H2B::mRFP* is expressed in vegetative nucleus. For better vegetative growth of plant, short‐day conditions in a growth chamber (light conditions: 8‐h light/16‐h dark; temperature: 22 ± 3°C and humidity: 55%) were used, while long‐day growth conditions (light conditions: 16‐h light/8‐h dark; temperature: 22 ± 3°C and humidity: 55%) were used to achieve flowering.

### 2.2 Mutagenesis and screen

Plants homozygous for the double transgene *MGH3p‐MGH3::eGFP* and *ACTIN11p‐H2B::mRFP* were selected for EMS mutagenesis. A total of 15,000 transgenic seeds were mutagenized by imbibition of .6% EMS for 12 h, .4% EMS for 8 h, and .3% EMS for 12 h (Kim et al., 2006). Well‐dried seeds were soaked in a 50‐ml plastic tube with 40 ml of 100 mM phosphate buffer (pH 7.5) at 4°C overnight and then treated with .3%, .4%, and .6% of EMS in a phosphate buffer for 8 h at room temperature with gentle shaking and finally washed thoroughly 20 times with ddH_2_O. The plants with phenotypes from M0 generation were separated and crossed back seven times with their parent plants (control). Reciprocal backcrosses with control enabled us to confirm that only male gametophyte is defective. After seven times reciprocal backcrosses, the target locus for mutant line was identified through second‐generation sequencing performed by BIONOVA Biotech Co., Ltd, Beijing. The raw reads of mutant samples obtained from sequencing company were aligned against the Col‐0 reference genome (TAIR10) using GenomeMapper. Consensus calling was performed by using SHORE consensus. SHOREmap v2.1 was used to predict mapping intervals for each of the bulked mapping populations. SNP variants identified in the resequencing analyses of the parental strains were filtered by SHOREmap create, and those passing the filtering were used as markers. SHOREmap backcross calculates the AF estimates at each marker position and determines mapping intervals according to the average AFs within sliding windows and the corresponding coefficients of variation. In addition to AFs, SHOREmap visualizes a different metric for the AFs. The AF score of SNP sites around .5 are kept for casual mutations.

### 2.3 Phenotypic analysis of mutants

Pollen grains of different developmental stages of *Arabidopsis* wild type as well as mutants were stained with a 4′,6‐diamidino‐2‐phenylindole (DAPI) solution (1 μg ml^−1^ DAPI and .1% Triton X‐100) for 5 min before observation. The viability of pollen grains was assessed using Alexander staining. The *paps1–4* mutant was transformed into quartet (qrt) mutant background to make tetrad pollen in which microspores fail to separate during pollen development (Preuss et al., 1994). For in vitro pollen germination, pollen was harvested from newly fully opened flowers and was placed onto pollen germination medium consisting of .01% (w/v) H_3_BO_3_, 1 mM MgSO_4_, 1 mM Ca (NO_3_)_2_, 1 mM CaCl_2_ and 18% (w/v) sucrose, pH 7.0, .8% (w/v) agar, and grown at 28°C in a growth chamber in the dark. The pollen grains were viewed with a laser scanning confocal microscope Zeiss LSM710 system, and images were collected by UltraView spinning‐disc confocal scanner unit (PerkinElmer). The excitation wavelength of 359 nm and the emission wavelength of 461 nm were used for DAPI fluorescent signals analysis. However, the excitation and detection wavelengths for GFP and RFP were 488 and 558 nm for excitation and 505–530 and 583 nm for detection, respectively. As the main purpose of this project was to explain the mechanism of cell fate determination, therefore we focused mainly on the isolation of mutants in which the normal expressions of male germline‐specific markers are defected. After EMS treatment, we also sequenced the whole regions of constructs to ensure that defects in the normal expression of markers do not result from any possible mutation in the original constructs.

### 2.4 Cloning and transformant generation

For *PAPS1p::PAPS1* complementation construct, the 2.5‐kb promoter and the full‐length coding region of *PAPS1* were cloned into a modified binary vector pCAMBIA1300‐bar (hygromycin resistance gene in pCAMBIA1300 has been replaced by Basta resistance gene). The binary vectors were introduced into *Agrobacterium tumefaciens* strain GV3101. The floral‐dip method was used for *Agrobacterium*‐mediated *Arabidopsis* transformation with minor modifications (Ali et al., 2022; Clough & Bent, 1998). The *paps1–4* mutant plants were transformed, and transformants were selected by using Basta antibiotic resistance plates. *MGH3p‐MGH3::eGFP*, *TIP5;1p‐MGH3::eGFP* and *DAW1p‐MGH3::eGFP* were used as sperm cell‐specific markers. For making *MGH3p‐MGH3::eGFP* construct, 1120 bps of MGH3 native promoter linked with male gamete histone 3 (MGH3) and green fluorescent protein (GFP) genes (2.67‐kb whole region) were cloned into a binary vector pCAMBIA1300. The same construct was modified by replacing *MGH3* promoter region with 1.2‐kb *TIP5;1* and 1.1‐kb *DAW1* promoters for creating *TIP5;1p‐MGH3::eGFP* and *DAW1p‐MGH3::eGFP* constructs, respectively. Vegetative nucleus‐specific marker *ACTIN11p‐H2B::mRFP* was constructed by fusing 940 bps of actin 11 (*ACT11*) promoter linked with Histone 2B (*H2B*) and red fluorescent protein (*RFP*) genes (2.17‐kb whole region) into a binary vector pCAMBIA1300. The construct was transformed into col. wild‐type plants.

## 3 RESULTS

### 3.1 *paps1–4* is male defective mutant

The isolated *paps1* mutant cannot transmit into progeny when used as a male parent in crosses with control female plant (Figure 1a). Although the mutant was completely pollen sterile, it exhibited normal vegetative and floral development. When used as the female parent in crosses with control plant, *paps1–4* produced viable seeds having siliques full seed‐set like those of the control plant, showing that the *paps1–4* mutation did not affect female fertility (Figure 1b). To analyze the developmental defects that caused male sterility in the mutant plant, pollen development and pollen tube germination in wild‐type and mutant plants were compared. The mutant pollen fails to germinate normal pollen tube (50% abnormal pollen tube germination, *n* = 1000) because of abnormal expression of *ACTIN11p‐H2B::mRFP* in sperm cells, leading to male defective gametophyte.

To know if *paps1–4* mutant phenotype is controlled by a single gene, crosses between mutant and control plant were made, and the Fl plants were leaved for autocrosses. Numbers of sterile plants and normal ones from both F1 and F2 populations were counted. The ratio of sterile plants to normal ones in both F1 (146/157) and F2 (autocross) (143/159) was about 1:1 (Table 1), which means that phenotype of *paps1–4* mutant plant is controlled by a single heterozygous nuclear gene.

**TABLE 1:** The *paps1–4* mutant causes pollen sterility

Female genotype	Male genotype	Progeny		
		*PAPS1*/*paps1* (mutant)	*PAPS1/PAPS1* (WT)	Ratio
*PAPS1*/*paps1*	*PAPS1/PAPS1*	146	157	1:1.07
*PAPS1/PAPS1*	*PAPS1/paps1*	0	300	0:1
*PAPS1/paps1*	*PAPS1/paps1*	143	159	1:1.11

### 3.2 PAPS1 is the casual factor for phenotype

**FIGURE 2:**
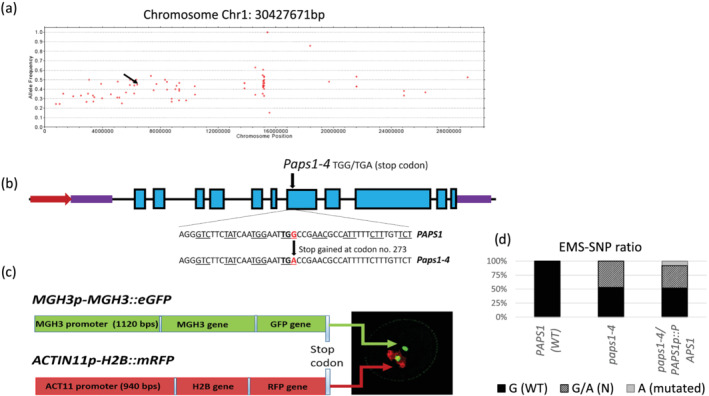
Map of *paps1–4*. Second‐generation sequencing showing all the EMS‐created mutation sites in red dots along with their allelic frequencies (AF) on chromosome 1. The *paps1–4* mutation site with AF value of .5 is marked with arrow. *Points in colors: AF (= alt divided by [alt + ref]) at markers. alt or ref: coverage of non‐reference or reference allele (a). Schematic representation of *PAPS1* gene structure and the position of mutant allele. The promoter is shown as a dark red arrow, introns is shown as thin black lines, exons as light blue rectangles, and 5′ and 3′ untranslated regions as purple rectangles. The position of the *paps1–4* point mutation (G to A change at nucleotide position 6189415 of chromosome 1) is indicated by the black arrow (b). Map of *Arabidopsis* double transgene *MGH3p‐MGH3::eGFP* and *ACTIN11p‐H2B::mRFP* used for EMS mutagenesis. Sperm cells‐specific marker *MGH3p‐MGH3::eGFP* consists of 1120 bps of MGH3 native promoter linked with male gamete histone 3 (MGH3) and green fluorescent protein (GFP) genes (2.67‐kb whole construct), whereas vegetative nucleus‐specific marker ACTIN11p‐H2B::mRFP consists of 940 bps of actin 11 (ACT11) promoter linked with Histone 2B (H2B) and red fluorescent protein (RFP) genes (2.17‐kb whole construct) (c). Sequencing mutated regions of 300 autocross *paps1–4* plants resulted in 158 plants with single G (WT) and 142 plants with double‐peak G/A (heterozygous condition linked with male gametophyte defective phenotype) with no homozygous peak for mutated A. However, homozygous peak for A was obtained when mutant plants were transformed with *PAPS1p::PAPS1* (d)

EMS mutagenesis creates many mutations at a DNA level. To clean unwanted mutations, *paps1–4* was continuously backcrossed with control plant. After seven times of backcrosses (BC), BC7 plants were separated into two groups, one group with phenotype and other group without phenotype. The leaves of 30 plants of each group were sent for second‐generation sequencing to BIONOVA sequencing company, Beijing. After getting the results from the company, the mutation sites for both groups were compared, and the values of expected allele frequencies (AFs) of .5 were detected for about three mutation sites on chromosome 1 (Figure 2a). Through continuous sequencing and phenotype comparison, the *paps1–4* mutation was linked with phenotype (Figure 2d), while the other two mutation sites segregated and did not co‐occur with phenotype. This is a point mutation of G nucleotide to A nucleotide where the codon substitution of TGG by TGA results in premature stop codon in exon 7 of the poly(A) polymerase PAPS1 (Figure 2b). Moreover, about 20 *paps1–4* mutant plants transformed with complementation construct (*PAPS1p::PAPS1*) were observed and found that the expression of complementation construct successfully recover the pollen defective phenotype of *paps1–4* mutant and attain the homozygous state for linked point mutation in the *paps1–4* plants due to the presence of functional copy of complementation construct (Figure 2d). This result shows that *PAPS1* is the actual target gene for the EMS‐created phenotype.

### 3.3 The expression of male germline‐specific promoter is defective in *paps1–4*

**FIGURE 3:**
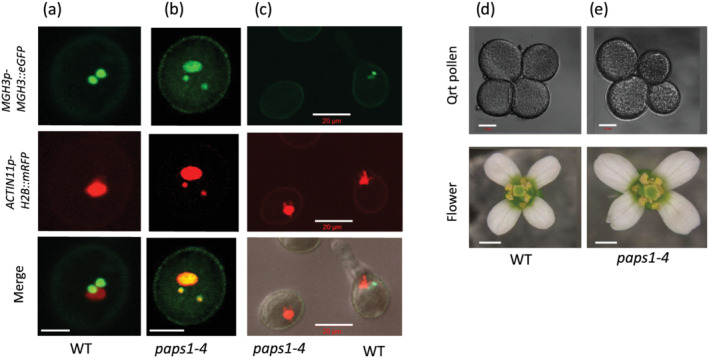
PAPS1 is required for sperm cell differentiation in plants. *Arabidopsis* pollen showing green fluorescence (*MGH3p‐MGH3::eGFP*; upper section) for sperm cells (SCs) and red fluorescence (*ACTIN11p‐H2B::mRFP*; middle section) for vegetative nucleus (VN) and merge (lower section) in wild‐type control plants (a). The *paps1–4* mutant showing abnormal green fluorescence (*MGH3p‐MGH3::eGFP;* upper section) for both SCs and VN and abnormal red signal (*ACTIN11p‐H2B::mRFP;* middle section) for both SCs and VN and merge (lower section) (b). In vitro pollen tube germination shows that WT pollen with normal fluorescence germinate successfully to produce pollen tube (c; WT), while *paps1–4* mutant pollen identified with abnormal red signal fails to germinate (c; *paps1–4*). The product of meiosis in qrt pollen showing all the four pollen of equal size (about 29 μm; d; upper section), while *paps1–4* mutant plant in qrt background produces two types of meiotic products, the pollen with the size nearly equal to the size of WT pollen (50%) and the pollen with the size smaller than the WT pollen (about 23 μm) (e; upper section). Images of *Arabidopsis* flowers in WT (d; lower) and *paps1–4* mutant plants (e; lower) showing altered flower growth. WT = Col‐0, bar scale 10 μm, or otherwise labeled, and 2 mm bottom row (d)

**FIGURE 4:**
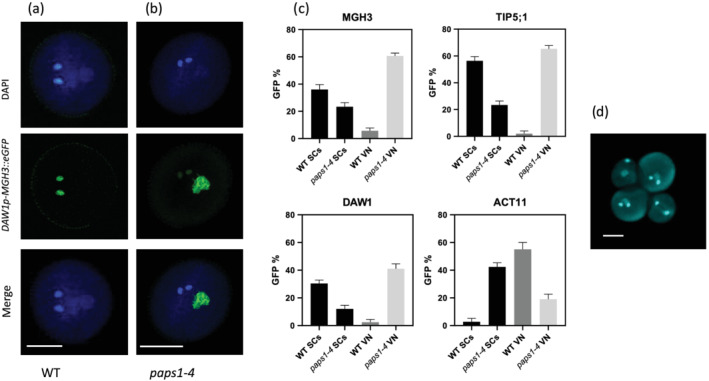
Sperm cell differentiation requires PAPS1‐dependent expression of the male germline‐specific genes. Examples of male germline‐specific *MGH3* gene promoter activity in WT and *paps1–4* pollen grains. Each panel shows a representative pollen grain under DAPI fluorescence (upper), GFP fluorescence (middle) and merge (lower). Only sperm cells in WT pollen show a GFP signal (a), while sperm cells in *paps1–4* show a residual level, but instead a strong GFP signal is shown by vegetative nucleus (b). Promoter activity of selected male germline‐specific genes *MGH3*, *TIP5;1* and *DAW1* and male germline‐nonspecific gene *ACT11*. GFP signal was recorded in single insert hemizygous lines in the *paps1*/+ background. The promoters of male germline‐specific genes show reduced activity in SCs while significantly increased activity in VN of *paps1–4* compared with wild‐type pollen grains. Each bar indicates the mean of three independent lines, and error bars display the SE (c). The DAPI fluorescence in qrt *paps1–4* plant showing two wild‐type tricellular pollen grains and two mutant‐type smaller bicellular pollen grains with delayed cell division (d). Bar scale 10 μm

*paps1–4* mutant was primarily identified based on defective expression of the red signal produced by male gamete‐nonspecific ACT11 promoter in SCs. In wild‐type tricellular mature pollen, the *MGH3p‐MGH3::eGFP* marker is only expressed in the sperm cells, while its expression is downregulated in the VN. Similarly, *ACTIN11p‐H2B::mRFP* expression is only limited to VN and its expression from sperm cells are strongly down regulated (Figure 2c and 3a). In *paps1–4* plant, mutant pollen is unable to downregulate the expression of male gamete‐nonspecific ACT11 promoter in SCs and male gamete‐specific MGH3 promoter in VN, resulting in strong red fluorescence for SCs and strong green fluorescence for VN, respectively (Figure 3b). About half (50%) of the pollen (*n* = 1000) of the mutant plant is showing the phenotype and is unable to germinate (Figure 3c). We also observed the cellular phenotype of *paps1–4* mutant pollen in qrt plant using light microscopy as well as DAPI staining. Mutant pollen was reduced in width (11 μm vs. 15 μm for wild‐type pollen; *p* < .001) and in length (21 μm vs. 28 μm for wild‐type pollen; *p* < .001) (Figure 3d,e, upper section). DAPI staining of pollen in qrt plants showed a 50% percentage of pollen with fewer cells (two cells) noticed than the three cells typical of wild‐type pollen (Figure 4d; smaller pollen vs. wild‐type pollen). This result show that pollen maturation in *paps1–4* mutant is delayed.

### 3.4 PAPS1 mediates sperm cell differentiation

The expression of male gamete‐specific MGH3 promoter in VN and male gamete‐nonspecific ACT11 promoter in SCs were strongly mis‐regulated in *paps1–4* mutant pollen. To know the exact role of PAPS1 in sperm cell differentiation, we observed the expression pattern of selective male germline specific promoters (*TIP5;1* and *DAW1)* in wild‐type and *paps1–4* mutant. Like *MGH3*, the expression pattern of both *TIP5;1* and *DAW1* was also defective in *paps1–4* as compared to wild type (Figure 4a,b). In *paps1–4*, the promoter activities of male gamete‐specific genes *MGH3*, *TIP5;1*, and *DAW1* were decreased in SCs but increased in VN (Figure 4c). However, the opposite case was found for male gamete‐nonspecific gene *ACT11*, and the activity of its promoter was increased in SCs but decreased in VN in *paps1–4* mutant. These data show that the activity of PAPS1 is essential for male gamete differentiation in *A. thaliana*.

## 4 DISCUSSION

The abnormality in *paps1–4* pollen developmental process appeared in tricellular stage, soon after pollen mitosis II (PMII). In control wild‐type plant, when the pollen is at bicellular stage, the GC expresses both green and red markers, while the VN does not express any of them. Soon after PMII, the green marker is still expressed in the resulting sperm cells; however, the expression of red marker is downregulated in the SCs but upregulated in VN (Borges et al., 2012; Figure 3a). The *paps1–4* mutant SCs fail to upregulate the expression of male gamete‐specific promoters and to downregulate male gamete‐nonspecific promoters. This shows that sperm cells have lost their identity as they cannot normally express the cell‐specific markers. The mutant pollen did not affect the second mitotic division, which can lead to production of germ cells with normal size and shape, suggesting that germ cells are produced normally, but in later stage they face some differentiation problem. However, observing *paps1–4* mutant pollen in qrt background, we found that the maturation of mutant pollen is delayed that is identified with two types of pollen at different stages of cell divisions arose from the same meiotic products of microspores. Half of the pollen are tricellular with sizes like wild‐type pollen grains, while the other half of the pollen are bicellular with smaller in sizes. To know whether the next cell division stops at bicellular stage in mutant pollen, or they may possibly gain their tricellular stage later, we examined the mutant pollen at the later stages of development and found that the tricellular state of pollen could be attained but somewhat delayed than the wild‐type; however, the differentiations of SCs are never achieved. These data show that PAPS1 does not affect pollen mitosis II but mediates sperm cell differentiation by influencing the expression of male germline‐specific regulon, an important activity to change the expression shift of proteins essential for gamete specificity and cell identity.

The importance of interaction among the cells of a pollen required for mediating SC differentiation is clarified by the presence of a cytoplasmatic bridge connecting the SCs as well as their positioning inside the VC of a pollen in tobacco and other species (Yu & Russell, 1993). Furthermore, small RNAs primarily originated from the VC were identified in SCs of *Arabidopsis* (Slotkin et al., 2009). Moreover, the promoter of ABA‐hypersensitive germination3 (AHG3) is transcriptionally active in the VC, whereas a translational fusion protein, AHG3‐GFP, driven by the same AHG3 promoter, was localized in SCs. These different localizations suggest that AHG3 transcripts or the AHG3 protein could move from the VC to SCs. These data show that both VC and SCs are linked and the transport of proteins between them is highly regulated.

The T‐DNA insertion mutant *paps1–3* was previously reported to exhibit similar phenotype of male gametophytic defect, as the mutant allele could not be transmitted through the pollen in reciprocal crosses (Vi et al., 2013). Furthermore, *paps1–3* mutant was previously found to show considerably reduced relative expression levels of the key genes for pollen differentiation like the LEUCINE‐RICH REPEAT/EXTENSIN 10 receptor kinase or SKU5 SIMILAR 13 (SKS13) and SKS14 (Zhang et al., 2019); however, a proper causal link of male gametophyte defect with SC differentiation was not established. Here, we found that *paps1*–4 mutant disturbs both male germline‐specific and germline‐nonspecific peptides and hence disrupts the whole regulating mechanism needed for SC differentiation. The undifferentiated SCs leading male gametophyte defective phenotype and the resulting pollen grains fail to germinate.

Like previously described *paps1–1* and *paps1–3* mutant phenotypes (Vi et al., 2013; Zhang et al., 2019), *paps1–4* mutant also exhibited opposite effects on flower and leaf growth, with larger flowers and smaller leaves. However, we also found another interesting phenotype of smaller pollen with inhibited pollen maturation. These opposite effects of PAPS1 on pollen (reduced growth), flower (increased growth), and leaf (reduced growth) indicate the different identities of these organs that determine a role for PAPS1 in the vital connection between growth pattern and organ identity. Previous study shows that it did not result in a global loss of poly(A) tails when PAPS1 activity was severely compromised in *paps1–1* seedlings grown at high temperatures (Vi et al., 2013), suggesting that PAPS1 influences the regulation of only specific peptides required for SC differentiation. Our results also confirm that loss‐of‐function mutant *paps1–4* does not bring a global loss but merely influences the upregulation of male germline specific proteins while the downregulation of male germline‐nonspecific peptides required for differentiation process.

## CONFLICT OF INTEREST

The authors declare no conflict of interest associated with the work described in this manuscript.

## ETHICS STATEMENT

We have obtained permissions to use *A. thaliana* Columbia‐0 (Col‐0) obtained from ABRC stock center (http://www.arabidopsis.org) and double transgenic line *MGH3p‐MGH3::eGFP* and *ACTIN11p‐H2B::mRFP* (Borges et al., 2012) for research purposes. All experimental research and field studies on plants including the collection of plant material were made by following relevant institutional, national, and international guidelines and legislation.

## AUTHOR CONTRIBUTIONS

I.A. and W.C.Y. designed the project. I.A. performed the experimental work. I.A., H.S., Z.U., A.A., and J.I. analyzed the data and wrote the article.

## Data Availability

The data that support the findings of this study are available from the corresponding author (Ali I) upon reasonable request.
